# Signatures of necroptosis-related genes as diagnostic markers of endometriosis and their correlation with immune infiltration

**DOI:** 10.1186/s12905-023-02668-7

**Published:** 2023-10-11

**Authors:** Xuezhen Wang, Qin Zheng, Man Sun, Luotong Liu, Huan Zhang, Weiwei Ying

**Affiliations:** 1 Department of Gynecology, Taizhou Hospital of Zhejiang Province Affiliated to Wenzhou Medical University, Taizhou, 317000 China; 2 School of Life Sciences, Jilin University, Changchun, 130012 China

**Keywords:** Endometriosis, Necroptosis, CIBERSORT, Immune microenvironment, Activated NK cells, M2 macrophage, MYO6, HOOK1

## Abstract

**Background:**

Endometriosis (EMS) occurs when normal uterine tissue grows outside the uterus and causes chronic pelvic pain and infertility. Endometriosis-associated infertility is thought to be caused by unknown mechanisms. In this study, using necroptosis-related genes, we developed and validated multigene joint signatures to diagnose EMS and explored their biological roles.

**Methods:**

We downloaded two databases (GSE7305 and GSE1169) from the Gene Expression Omnibus (GEO) database and 630 necroptosis-related genes from the GeneCards and GSEA databases. The limma package in Rsoftware was used to identify differentially expressed genes (DEGs). We interleaved common differentially expressed genes (co-DEGs) and necroptosis-related genes (NRDEGs) in the endometriosis dataset. The DEGs functions were reflected by gene ontology analysis (GO), pathway enrichment analysis, and gene set enrichment analysis (GSEA). We used CIBERSORT to analyze the immune microenvironment differences between EMS patients and controls. Furthermore, a correlation was found between necroptosis-related differentially expressed genes and infiltrating immune cells to better understand the molecular immune mechanism.

**Results:**

Compared with the control group, this study revealed that 10 NRDEGs were identified in EMS. There were two types of immune cell infiltration abundance (activated NK cells and M2 macrophages) in these two datasets, and the correlation between different groups of samples was statistically significant (*P* < 0.05). MYO6 consistently correlated with activated NK cells in the two datasets. HOOK1 consistently demonstrated a high correlation with M2 Macrophages in two datasets. The immunohistochemical result indicated that the protein levels of MYO6 and HOOK1 were increased in patients with endometriosis, further suggesting that MYO6 and HOOK1 can be used as potential biomarkers for endometriosis.

**Conclusions:**

We identified ten necroptosis-related genes in EMS and assessed their relationship with the immune microenvironment. MYO6 and HOOK1 may serve as novel biomarkers and treatment targets in the future.

**Supplementary Information:**

The online version contains supplementary material available at 10.1186/s12905-023-02668-7.

## Introduction

Endometriosis occurs when endometrial glands and stroma appear outside the uterine cavity [1]. The predicted prevalence of this disease at reproductive age is 10% [2]. Endometriosis reduces women's health-related quality of life (HRQOL), resulting in impairments in physical functioning, diminished social life, difficulties in intimate relationships, and decreased productivity. The endometriosis etiology is complex, involving multiple genetic and environmental risk factors. Although the endometriosis pathogenesis is relatively unclear, it is believed to be caused by retrograde menstruation leading to exfoliated endometrium implantation. However, there are limited studies on the development of endometriosis, endometrial-peritoneal attachments, and invasion. Endometriosis is difficult to diagnose without biomarkers to detect or rule out [3]. Biomarkers and novel therapies targeting the diverse physiological mechanisms associated with the onset, progression, and persistence of endometriosis symptoms are urgently required [4].

Necroptosis, also known as necroptosis, is a receptor-interacting serine/threonine protein kinase 1 (RIPK1), RIPK3, and mixed lineage kinase domain-containing pseudo kinase (MLKL), but is not Caspase-1 dependent [5]. Necroptosis has been implicated as a critical cell death pathway in cancers, Alzheimer's, other neurodegenerative diseases, and virus-infected cells [6]. Some studies have identified necroptotic modulators as possible prognostic biomarkers for cancer and certain diseases [7, 8]. Day et al. [9] found that BMI1 in ovarian cancer can participate in the PINK1-Park2-dependent mitochondrial pathway and induce a new type of non-apoptosis cell death mediated by necroptosis. Endometriosis severity is related to apoptosis, which usually destroys ectopic and heterotopic endometrial cells before forming necrotic tissue, thus inhibiting their migration and accumulation [10, 11]. Moreover, apoptotic mechanisms in the cytoplasm and cellular inflammasomes can further interact with ERβ-induced immune surveillance. However, the mechanism and function of necroptosis in endometriosis progression remains unclear.

Growing evidence suggests that the immune system is vital to the pathophysiology and symptoms of EM. Immune cells such as natural killer (NK) cells, macrophages, neutrophils, and CD4 T helper cells are dysregulated in women with EM [12, 13]. Immune-related mechanisms have been described as involved in the pathophysiology and symptomatology of EMS by contributing to the survival and persistence of endometriosis lesions [14]. Immune dysfunction is associated with the implantation, proliferation, and apoptosis of ectopic endometrium. However, in women with endometriosis, it is unclear which subtypes of immune cells are presented in the ectopic endometrium. Analyzing the relationship between necroptosis and immune infiltration may help to explore unknown mechanisms. Recently, in a meta-analysis of transcriptomes using the xCell algorithm, immune profiles in eutopic endometriosis and stages I–II and III–IV endometriosis were significantly different, regardless of the hormone [12]. Therefore, exploring immune mechanisms in endometriosis is key to elucidate their role in endometriosis pathogenesis and generating unique insights for developing preventive and therapeutic strategies, innovative noninvasive diagnostic methods, and targeted therapies.

This study explored potential biomarkers of endometriosis and their biological effects on the pathogenesis of endometriosis. We used the gene expression datasets GSE11691 and GSE7305 associated with normal and ectopic endometrium, respectively, which were extracted from the Gene Omnibus (GEO) database. Differential genes were screened and intersected with necroptosis-related genes. Subsequently, the immune microenvironment was compared between endometriosis patients and controls using CIBERSORT, and immune cell association was calculated with NRDEGs for the first time. We performed a bioinformatics analysis of endometriosis to elucidate the endometriosis pathogenesis further.

## Materials and methods

### Downloading data

We downloaded endometriosis-related datasets GSE7305 [15] and GSE1169 from GEO database [16] through R package GEOquery [17]. GSE7305 dataset, which comes from Homo Sapiens and the data platform is GPL570, contains 20 samples, including 10 endometriosis and 10 normal samples. Moreover, GSE11691 dataset, from Homo Sapiens and data platform GPL96, contained 18 samples, nine of which were endometriosis and nine normal. All samples from two datasets were included in the study.

The GeneCards database [18] provides comprehensive information on human genes. Necroptosis-related genes were obtained using the word "necroptosis" as the search keyword in GeneCards and GSEA databases [19]. A total of 630 necroptosis-related genes were obtained after merging and deduplication (Table S1).

### Analysis of differentially expressed genes associated with endometriosis

To identify possible mechanisms and pathways associated with differential gene expression in endometriosis, R package limma was used to standardize the datasets GSE7305 and GSE11691, and the expression profile data after processing were analyzed differently. DEGs between different subgroups were obtained from two endometriosis datasets, |logFC|> 0.5 and *P*.adj < 0.05, which were used as standards to further screen the DEGs involved in this study. Among them, genes with logFC > 0.5 and p.adj < 0.05 were upregulated DEGs. Genes with logFC < -0.5 and *p*.adj < 0.05 were downregulated DEGs.

To obtain the necroptosis-related differentially expressed genes (NRDEGs) of endometriosis, we first intersected all differentially expressed genes with |logFC|> 0.5 and *P*.adj < 0.05 obtained from the differential analysis of dataset GSE7305 and dataset GSE11691 and plotted the Venn diagram to visualize the common differentially expressed genes of the dataset. Venn diagrams were then used to visualize the co-DEGs intersection and necroptosis-related genes between the two datasets. The difference analysis results were displayed by volcano map using R package ggplot2, and a heatmap was drawn using R package pheatmap.

### Functional enrichment analysis

Gene ontology (GO) [20, 21] analysis is a common method for large-scale functional enrichment studies, including biological processes (BP), molecular functions (MF), and cellular components (CC). The R package clusterprofiler [22] was used for GO analysis of NRDEGs. To qualify for entry, the screening criteria were a *P*-value of 0.05 and an FDR value (Q value) of 0.05, which was considered statistically significant. The *P*-value correction method was Benjamini-Hochberg (BH).

### Gene set enrichment analysis

Gene Set Enrichment Analysis (GSEA) [23] was used to evaluate the correlation between genes from a predefined Gene Set and phenotypes in the Gene Table to measure its phenotypic contribution. In this study, genes in GSE7305 dataset (Table 2) and GSE11691 dataset (Table 3) were divided into high- and low-phenotypic correlations according to the ranking of the phenotypic correlation degree. The R package clusterProfiler enriched and analyzed all DEGs in the two groups. Following are the parameters used in this GSEA: The seed was 2020, the number of calculations was 1000, the minimum number of genes in each gene set was 10, and the maximum number of genes was 500. The correction method for the *P*-value was Benjamini-Hochberg (BH). The Molecular Signatures Database (MSigDB) [24] provided the C2.cp.v7.2. symbol gene set and the screening criteria for significant enrichment were *P* < 0.05 and FDR value (Q value) < 0.25.

### PPI, mRNA-miRNA, mRNA-TF, mRNA-Drug interaction network

Protein–protein interactions (PPI) are composed of individual proteins. The STRING database [25] searches for interactions between predicted and known proteins. This study used the STRING database to construct a protein–protein interaction network related to differentially expressed genes (minimum required interaction score: low confidence (0.150). The PPI network model was visualized using Cytoscape [26] software (version 3.9.1).

Using the Starbase (Version 3.0) database [27], we searched for miRNA targets by analyzing the experimental data generated by CLIP-seq and degradation groups, providing various visual interfaces for exploring miRNA targets. The database contains abundant miRNA-ncRNA, miRNA-mRNA, miRNA-RNA, and RNA-RNA data. miRDB database [28] was used for miRNA target-gene prediction and functional annotation. We used the Starbase and miRDB databases to predict miRNAs interacting with key genes (mRNAs) and then used the intersection part of the results from the two databases to draw the mRNA-miRNA interaction network using Cytoscape software.

CHIPBase database [29] (version 2.0) (https://rna.sysu.edu.cn/chipbase/) from the DNA binding protein. ChIP-seq data identified thousands of combining base sequence matrices and binding sites and predicted millions of transcriptional regulatory relationships between transcription factors (TFs) and genes. HTFtarget database [30] (http://bioinfo.life.hust.edu.cn/hTFtarget) is a comprehensive database of human TFs and their targets. We searched for TFs that bind to key genes using CHIPBase and hTFtarget databases, extracted the intersection parts, and plotted the mRNA-TF interaction network using Cytoscape software.

We also predicted the direct and indirect drug targets of NRDEGs through CTD (Comparative Toxicogenomics Database) [31] explored the interaction between NRDEGs and drugs, and used Cytoscape software to visualize the mRNA-drug interaction network.

### Expression differences, chromosomal localization, and functional similarity analysis of NRDEGs

We analyzed NRDEG expression in endometriosis datasets GSE7305 and GSE11691. To analyze NRDEGs localization in 24 pairs of chromosomes, we first used UCSC database (http://genome.ucsc.edu/) [32] to determine the start and stop sequences of NRDEGs. Subsequently, R package RCircos [33] was used to map the chromosome localization. GoSemSim [34] is an R software package used to calculate the semantic similarity between gene products, gene clusters, and GO terms. To analyze the functional correlations among key genes, R package GOSemSim was used to calculate the functional correlations of key genes.

### Immunohistochemistry

All the endometriosis patient tissues were obtained from Taizhou Hospital of Zhejiang Province affiliated to Wenzhou Medical University. Informed consent was obtained from all participating patients and the study was approved by the ethics committee of biomedical research involving humans (Approval No. K20230901). In detail, tissues from patients with endometriosis were first fixed in 10% formalin and paraffin embedded. Tissue sections were dewaxed and rehydrated in xylene and graded alcohol solutions. High-temperature antigen extraction was then performed in citrate buffer (pH 6). Primary antibodies were incubated overnight at 4 °C, and secondary antibodies were incubated at room temperature for 30 min. After washing, sections were stained with a DAB peroxidase substrate kit until the desired intensity was achieved.

### Analysis of immune infiltration

CIBERSORTx [35] is an immune infiltration analysis algorithm based on linear support vector regression that deconvolves the transcriptome expression matrix to estimate the composition and immune cell abundance in mixed cells. We uploaded the data gene expression matrix to CIBERSORTx online website (https://cibersortx.stanford.edu/), combined with Homo sapiens gene matrix (Homo sapiens) characteristics, and screened for immune cell enrichment scores greater than zero. Finally, specific immune cell infiltration abundance matrix results were obtained and demonstrated. The difference in the proportion of immune cells between endometriosis samples (group: endometriosis) and normal samples (group: normal) in the endometriosis dataset was calculated using the Wilcoxon test, and a *P*-value < 0.05 was considered statistically different. The correlation of immune cells between different groups was calculated using Spearman and visualized by R package ggplot2. We then combined the gene expression matrix of the dataset to calculate the correlation between immune cells and NRDEGs and drew a correlation heatmap using R package pheatmap.

## Results

### Technical roadmap

Figure 1.

**Fig. 1:**
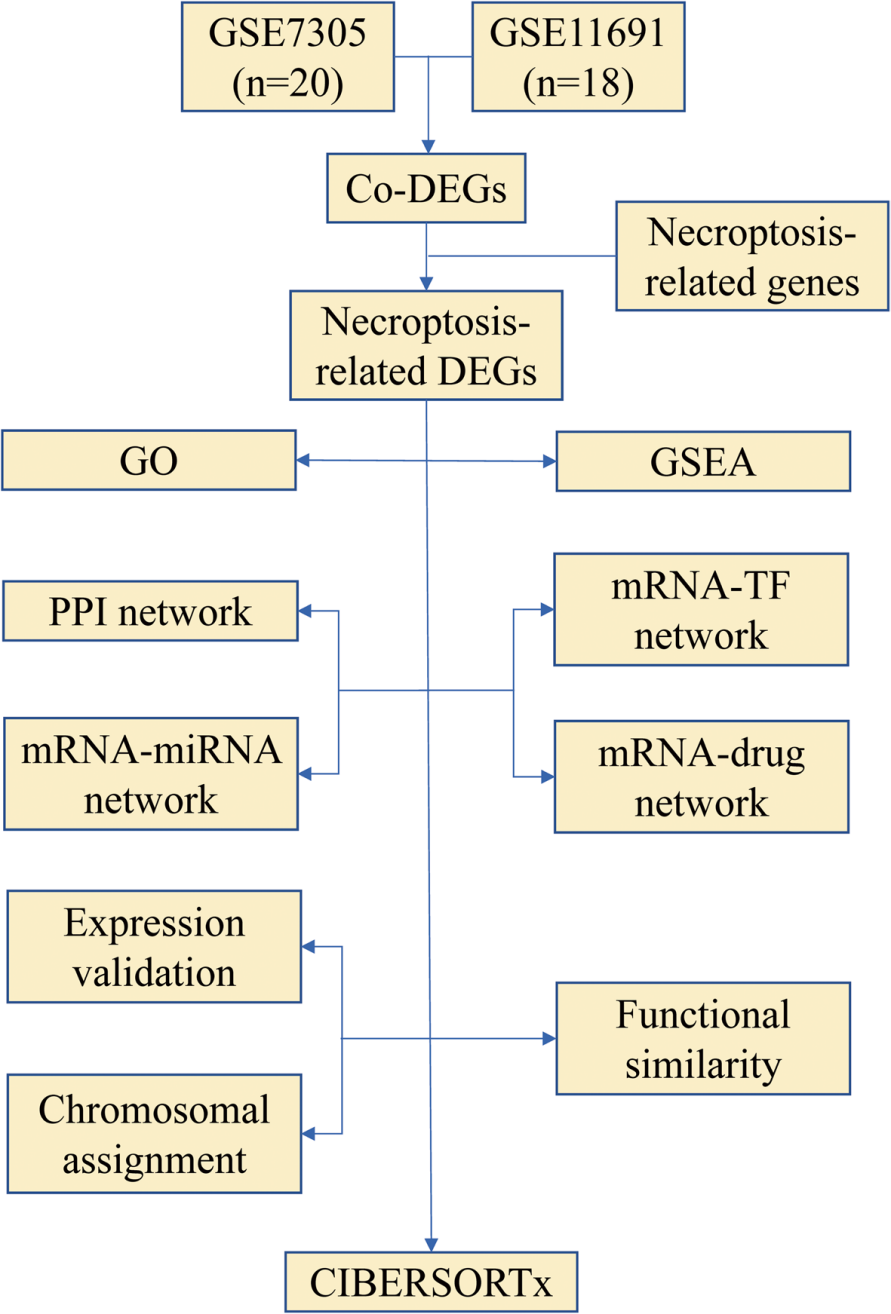
Technology roadmap

### Analysis of endometriosis-related differentially expressed genes

**Fig. 2:**
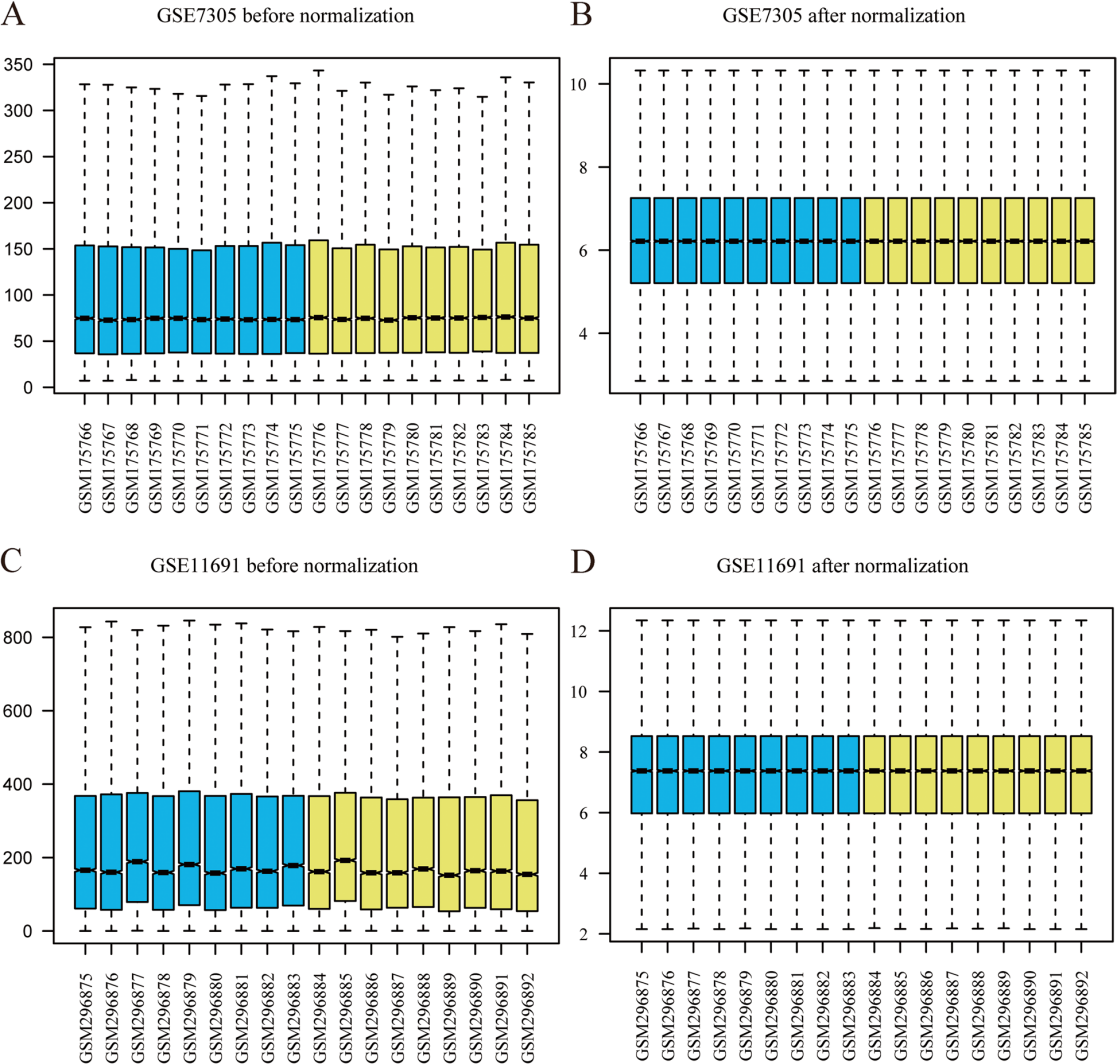
Presentation of results *of standardized processing of the endometriosis dataset. Endometriosis dataset GSE7105 is displayed in the data box before (**A**) and after (**B**) standardized treatment. Endometriosis dataset GSE1169 is displayed in the data box before (**C**) and after (**D**) standardized treatment

**Fig. 3:**
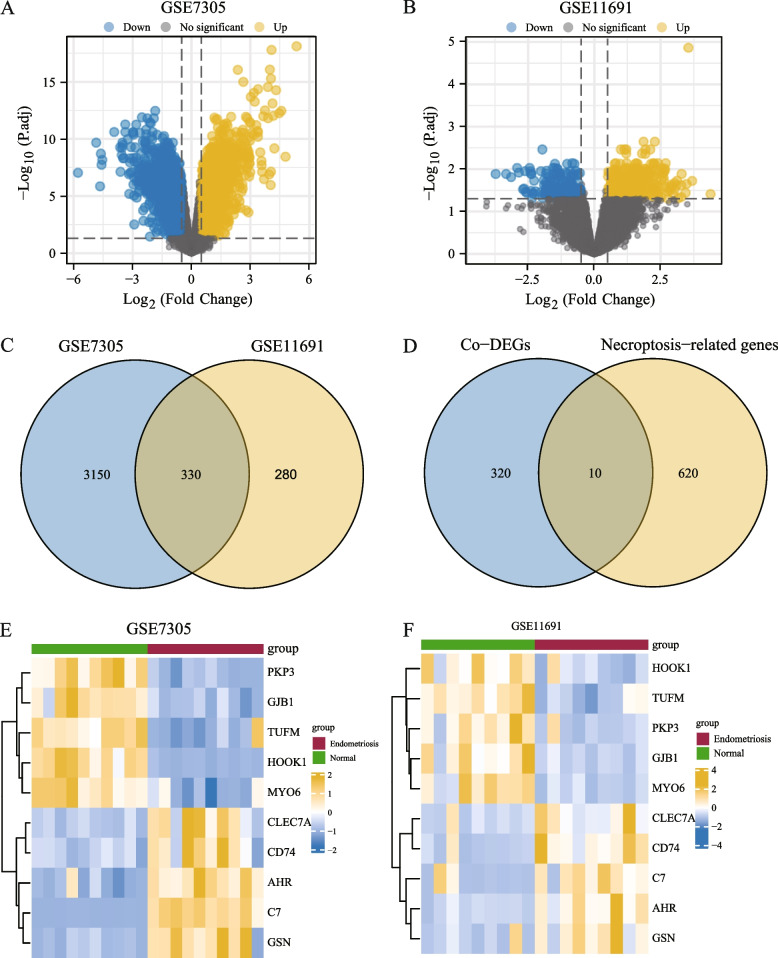
Endometriosis-related differentially expressed genes analysis. Volcano map of differentially expressed genes analysis between endometriosis (group: endometriosis) and normal endometrial tissue (group: normal) in GSE7305 dataset (**A**) and GSE11691 dataset (**B**). (**C**) Venn diagram of differentially expressed genes in the GSE7305 dataset and GSE11691 dataset. (**D**) Venn diagram of co-DEGs and necroptosis-related genes in the dataset. (**E**) Complex numerical heatmaps of NRDEGs in GSE7305 dataset. (**F**) GSE11691 dataset. Co-DEGs, common differentially expressed genes. NRDEGs, necroptosis-related differentially expressed genes

Using the limma package, we first normalized the expression profile data of the endometriosis datasets, GSE7305 and GSE11691. The data distribution before and after standardized treatment is revealed in a box plot (Figs. 2A–D). We found that the data after standardized treatment tended to be consistent in their expression levels. To analyze the gene expression values in endometriosis dataset samples (group: endometriosis) relative to normal samples (group: endometriosis), we used R package limma to analyze the differences between datasets GSE7305 and GSE11691 and obtained the differentially expressed genes from the two datasets. The results were as follows: Dataset GSE7305 got 20,247 DEGs, of which 3480 genes met the | logFC |> 0.5 and *P*.adj < 0.05. At this threshold, the number of highly expressed (low expression in the normal group, positive logFC, upregulated genes) in the endometriosis group was 1760, and the number of low expressed (high expression in the normal group, negative logFC, downregulated genes) in the endometriosis group was 1720. A volcano map was constructed based on the analysis results of this dataset (Fig. 3A). The GSE11691 got 12,376 DEGs, 610 genes met the | logFC |> 0.5 and *P*.adj < 0.05, and under the threshold, the number of highly expressed genes (low expression in the normal group, positive logFC, upregulated genes) in the endometriosis group was 396. The number of genes with low expression (high expression in the normal group, negative logFC, downregulated genes) in the endometriosis group was 214. We drew a volcano map based on the differential analysis results from the GSE11691 dataset (Fig. 3B).

To obtain the NRDEGs, we intersected the DEGs from GSE7305 and GSE11691 with |logFC|> 0.5 and *P*.adj < 0.05, 330 common differentially expressed genes (co-DEGs) of the endometriosis dataset were obtained, and a Venn diagram was drawn (Fig. 3C). We also examined the intersection between the co-DEGs and necroptosis-related genes using the endometriosis dataset. Ten NRDEGs from the endometriosis dataset were obtained, and a Venn diagram was drawn (Fig. 3D), which were C7, HOOK1, PKP3, AHR, TUFM, GJB1, GSN, MYO6, CLEC7A, and CD74. According to the results obtained from the Venn diagram, the expression differences of 10 NRDEGs in the GSE7305 (Fig. 3E) and GSE11691 datasets (Fig. 3F) among different sample groups were analyzed, and the R package pheatmap was used to draw a heatmap to display the analysis results (Figs. 3E and F). The results demonstrated that PKP3, GJB1, HOOK1, TUFM, and MYO6 were upregulated (low expression in the normal group, positive logFC, yellow in the figure), whereas C7, AHR, GSN, CLEC7A, and CD74 were downregulated (high expression in the normal group, blue in the figure, logFC is negative).

### Functional enrichment analysis

**Fig. 4:**
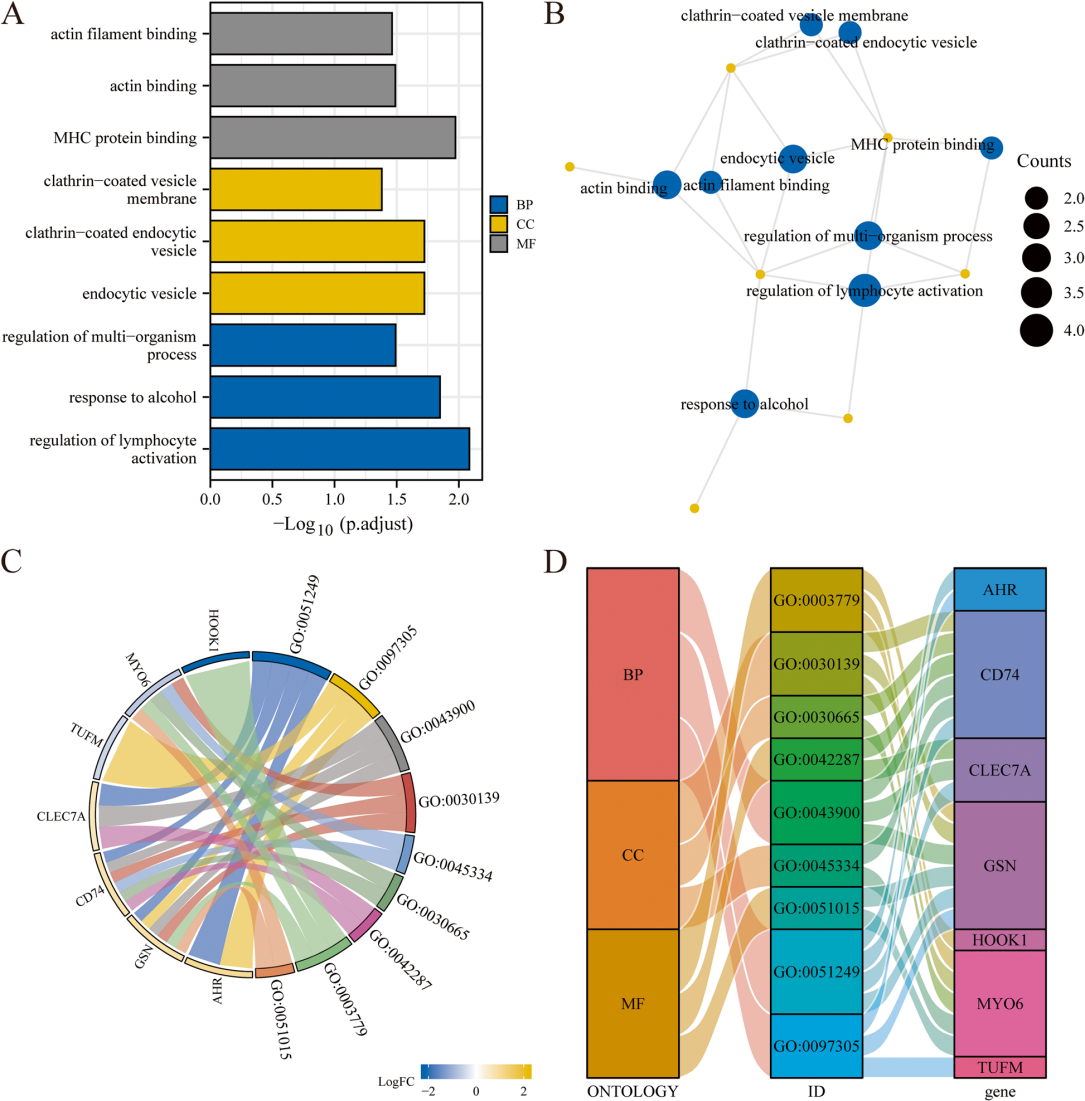
Functional Enrichment Analysis of NRDEGs (GO). (**A**) Bubble diagram of GO functional enrichment analysis results of NRDEGs. Network diagram of GO functional enrichment analysis results of NRDEGs. In the circular network diagram (**B**), yellow dots represent specific genes, and blue circles represent specific pathways. (**C**) Chord plots of GO functional enrichment combined with logFC analysis results of NRDEGs. (**D**) Sankey diagram showing the results of GO functional enrichment analysis of NRDEGs. GO, gene ontology; BP, biological process; CC, cell component; MF, Molecular Function. The screening criteria for GO enrichment items were *P* value < 0.05 and FDR value (Q value) < 0.05. NRDEGs, necroptosis-related differentially expressed genes

To evaluate the biological processes, molecular functions, cell components, biological pathways, and endometriosis of 10 NRDEGs (C7, HOOK1, PKP3, AHR, TUFM, GJB1, GSN, MYO6, CLEC7A, and CD74), we first conducted GO (gene ontology) analysis for NRDEGs (Table 1). The screening criteria for enrichment items were *P* value < 0.05 and FDR value (Q value) < 0.05, which were considered statistically significant. The results demonstrated that 10 NRDEGs (C7, HOOK1, PKP3, AHR, TUFM, GJB1, GSN, MYO6, CLEC7A, and CD74) were mainly enriched in biological processes, such as regulation of lymphocyte activation in endometriosis, response to alcohol, regulation of multi-organism processes, and cellular components (CC), such as endocytic vesicles, clathrin-coated endocytic vesicles, and clathrin-coated vesicle membranes. It was also enriched in molecular function (MF), including MHC protein binding, actin binding, and actin filament binding. We demonstrated GO functional enrichment analysis results using a bubble diagram (Fig. 4A). Furthermore, GO gene functional enrichment analysis results are presented in the network diagram (Fig. 4B). Subsequently, we conducted a GO enrichment analysis of the 10 NRDEGs combined with logFC. Moreover, based on the enrichment analysis, we calculated each molecule's corresponding Z score by the molecule's logFC value in the differential analysis result of the provided molecule in the endometriosis dataset. We present the GO enrichment analysis results of combined logFC using a chord diagram (Fig. 4C). The GO enrichment analysis results are displayed in the form of a Sankey diagram (Fig. 4D), including BP, CC, MF (biological process, cellular component, and molecular function), and their corresponding function or pathway ID (ID) and category ID (ID) including the relationship between the gene names (gene).

**Table 1:** GO enrichment analysis results of necroptosis-related differentially expressed genes

Ontology	ID	Description	GeneRatio	BgRatio	*p*-value	*p*.adjust	qvalue
BP	GO:0051249	regulation of lymphocyte activation	4/10	485/18670	8.34e-05	0.008	0.004
BP	GO:0097305	response to alcohol	3/10	233/18670	2.16e-04	0.014	0.007
BP	GO:0043900	regulation of multi-organism process	3/10	405/18670	0.001	0.032	0.016
CC	GO:0030139	endocytic vesicle	3/10	303/19717	3.98e-04	0.019	0.010
CC	GO:0045334	clathrin-coated endocytic vesicle	2/10	63/19717	4.45e-04	0.019	0.010
CC	GO:0030665	clathrin-coated vesicle membrane	2/10	115/19717	0.001	0.042	0.021
MF	GO:0042287	MHC protein binding	2/9	40/17697	1.78e-04	0.011	0.004
MF	GO:0003779	actin binding	3/9	431/17697	0.001	0.032	0.013
MF	GO:0051015	actin filament binding	2/9	198/17697	0.004	0.034	0.014

*GO* Gene ontology, *BP* Biological process, *CC* Cell component, *MF* Molecular function

### Gene set enrichment analysis

**Fig. 5:**
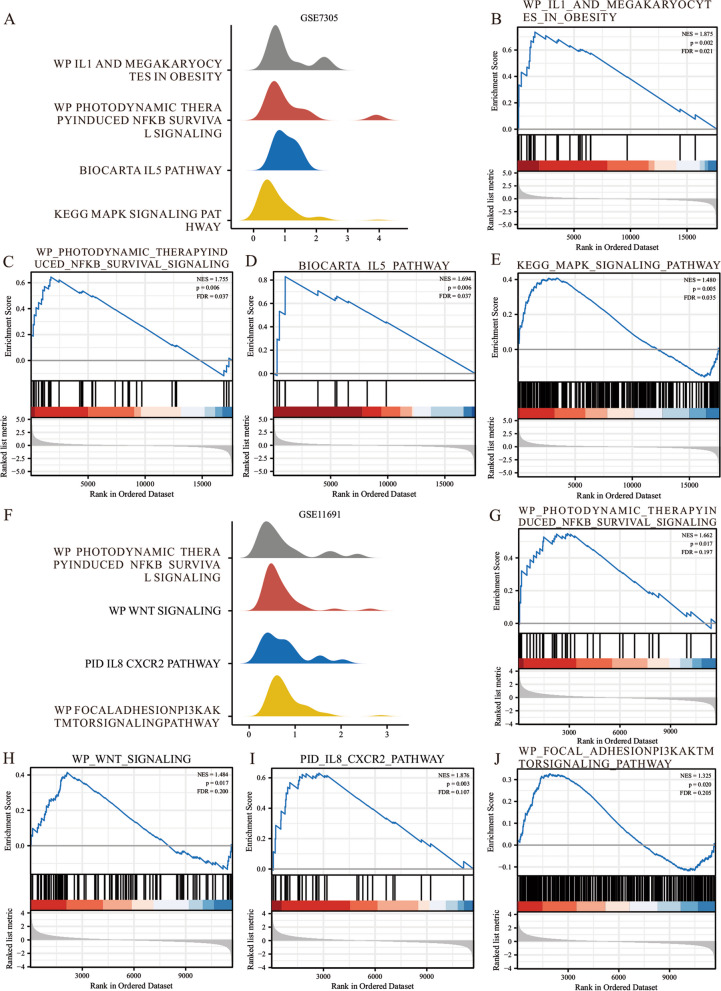
GSEA enrichment analysis of the endometriosis dataset. (**A**) Four main biological characteristics of GSEA enrichment analysis for GSE7305 dataset. GSE7305 is significantly enriched in WP_IL1_AND_MEGAKARYOCYTES_IN_OBESITY (**B**), WP_PHOTODYNAMIC_THERAPYINDUCED_NFKB_SURVIVAL_SIGNALING (**C**), BIOCARTA_IL5_PATHWAY (**D**), KEGG_MAPK_SIGNALING_PATHWAY [36–38] (**E**), and other pathways (**F**). Four main biological characteristics of GSEA analysis in GSE11691 dataset. GSE11691 dataset is significantly enriched in WP_PHOTODYNAMIC_THERAPYINDUCED_ NFKB_SURVIVAL_SIGNALI NG (**G**), WP_WNT_SIGNALING (**H**), WP_FOCAL_ADHESIONPI3KAKTMTO RSIGNALING_PATHWAY PID_IL8_CXCR2_PATHWAY (**I**), (**J**). The significant enrichment screening criteria for GSEA enrichment analysis were *P* < 0.05 and FDR value (Q value) < 0.25. GSEA, Gene Set Enrichment Analysis

To determine the impact of the expression levels of all genes related to endometriosis metabolism on the occurrence of endometriosis, we evaluated the gene expression profile and the biological processes involved in GSE7305 dataset (Fig. 5A) and GSE11691 dataset (Fig. 5F) by GSEA (Gene Set Enrichment Analysis) enrichment analysis, respectively. Links between cellular components and their molecular functions. *P* < 0.05 and FDR value (Q value) < 0.25 were considered significant enrichment screening criteria. The results displayed that differentially expressed genes in dataset GSE7305 were significantly enriched in IL1 and megakaryocytes in obesity (Fig. 5B), photodynamic therapy-induced NFKB survival signaling (Fig. 5C), the IL5 pathway (Fig. 5D), MAPK signaling pathway (Fig. 5E), and other pathways (Figs. 5A–E, Table 2). However, differentially expressed genes in dataset GSE11691 were significantly enriched in photodynamic therapy-induced NFKB survival signaling (Fig. 5G), Wnt signaling (Fig. 5H), IL8 CXCR2 pathway (Fig. 5I), and focal adhesion PI3K-AKT mTOR signaling pathway (Fig. 5J), and other pathways (Figs. 5F–J, Table 3).

**Table 2:** GSEA analysis of dataset GSE7305

Description	setSize	enrichmentScore	NES	*p*-value	*p*.adjust
REACTOME_COMPLEMENT_CASCADE	56	0.822144048	2.431471926	0.001862197	0.028091787
KEGG_COMPLEMENT_AND_COAGULATION_CASCADES	67	0.762017649	2.333823306	0.001841621	0.028091787
WP_HUMAN_COMPLEMENT_SYSTEM	92	0.709058749	2.296694499	0.001795332	0.028091787
WP_COMPLEMENT_AND_COAGULATION_CASCADES	57	0.776932609	2.293240512	0.001879699	0.028091787
WP_COMPLEMENT_ACTIVATION	20	0.883248685	2.184014586	0.001984127	0.028091787
REACTOME_INITIAL_TRIGGERING_OF_COMPLEMENT	23	0.845953791	2.156923481	0.001964637	0.028091787
KEGG_SYSTEMIC_LUPUS_ERYTHEMATOSUS	50	0.739155934	2.155146117	0.001855288	0.028091787
BIOCARTA_COMP_PATHWAY	17	0.900396432	2.141644522	0.002	0.028091787
WP_TYROBP_CAUSAL_NETWORK	59	0.705926715	2.111012452	0.001821494	0.028091787
BIOCARTA_LAIR_PATHWAY	17	0.873581328	2.077863261	0.002	0.028091787
WP_CELLS_AND_MOLECULES_INVOLVED_IN_LOCAL_ACUTE_INFLAMMATORY_RESPONSE	17	0.873581328	2.077863261	0.002	0.028091787
WP_IL1_AND_MEGAKARYOCYTES_IN_OBESITY	24	0.736409022	1.875075348	0.001972387	0.028091787
WP_PHOTODYNAMIC_THERAPYINDUCED_NFKB_SURVIVAL_SIGNALING	35	0.644242533	1.754669341	0.005859375	0.048273954
BIOCARTA_IL5_PATHWAY	10	0.829191458	1.693843996	0.006012024	0.048273954
KEGG_MAPK_SIGNALING_PATHWAY	254	0.408313605	1.480258186	0.005016722	0.045404266

*GSEA* Gene Set Enrichment Analysis

**Table 3:** GSEA analysis of dataset GSE11691

Description	setSize	enrichmentScore	NES	*p*-value	*p*.adjust
WP_TYROBP_CAUSAL_NETWORK	50	0.701769525	2.23878553	0.001658375	0.11042735
REACTOME_SMOOTH_MUSCLE_CONTRACTION	35	0.722865333	2.195371633	0.001644737	0.11042735
NABA_CORE_MATRISOME	188	0.556594657	2.179478164	0.00136612	0.11042735
REACTOME_ELASTIC_FIBRE_FORMATION	38	0.688839129	2.119760258	0.001628664	0.11042735
REACTOME_MOLECULES_ASSOCIATED_WITH_ELASTIC_FIBRES	33	0.692726707	2.086001585	0.001644737	0.11042735
NABA_ECM_GLYCOPROTEINS	127	0.552642372	2.048692518	0.001490313	0.11042735
REACTOME_MUSCLE_CONTRACTION	175	0.52131301	2.020357098	0.001371742	0.11042735
KEGG_SYSTEMIC_LUPUS_ERYTHEMATOSUS	49	0.631792413	2.009084444	0.001666667	0.11042735
REACTOME_CHEMOKINE_RECEPTORS_BIND_CHEMOKINES	52	0.618962401	1.988331531	0.001658375	0.11042735
REACTOME_ECM_PROTEOGLYCANS	70	0.581720599	1.98573096	0.001587302	0.11042735
REACTOME_EXTRACELLULAR_MATRIX_ORGANIZATION	255	0.492682565	1.981455275	0.001329787	0.11042735
PID_IL8_CXCR2_PATHWAY	32	0.631225305	1.875950683	0.003372681	0.118114144
WP_PHOTODYNAMIC_THERAPYINDUCED_NFKB_SURVIVAL_SIGNALING	34	0.549039654	1.662008258	0.016583748	0.217999152
WP_WNT_SIGNALING	95	0.41447078	1.483747047	0.017160686	0.221492947
WP_FOCAL_ADHESIONPI3KAKTMTORSIGNALING_PATHWAY	278	0.326507177	1.324520451	0.019556714	0.227236181

*GSEA* Gene Set Enrichment Analysis

### Construction of PPI, mRNA-miRNA, mRNA-TF, and mRNA-drug regulatory networks

**Fig. 6:**
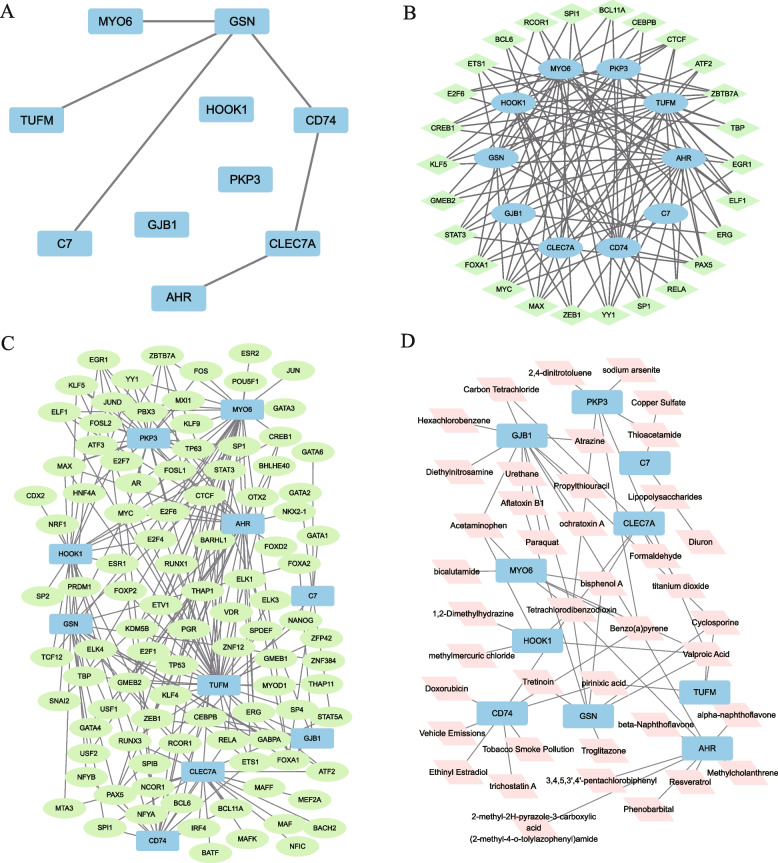
PPI, TF-mRNA, mRNA-miRNA, and mRNA-drug regulatory networks. (**A**) NRDEGs PPI Network. (**B**) mRNA-miRNA regulatory network: blue oval is mRNA, and green diamond is miRNA. (**C**) mRNA-TF regulatory network: the blue rectangle is mRNA; the green oval is TF. (**D**) mRNA-drug regulatory network: the blue rectangle is mRNA; the pink diamond is a drug. TF, Transcription factor. PPI, protein–protein interaction; NRDEGs, necroptosis-related differentially expressed genes

First, protein–protein interaction analysis was conducted using STRING database with a minimum required interaction score greater than 0.150. Low confidence (0.150) was used as the standard to construct a PPI network of 10 NRDEGs (C7, HOOK1, PKP3, AHR, TUFM, GJB1, GSN, MYO6, CLEC7A, and CD74). The interactions were visualized using Cytoscape software (Fig. 6A). There were only seven NRDEGs in the PPI network: C7, AHR, TUFM, GSN, MYO6, CLEC7A, and CD74. Second, miRNAs related to NRDEGs were obtained from StarBase and miRDB databases. To visualize the mRNA-miRNA regulatory network, Cytoscape was applied (Fig. 6B), which contained 10 mRNA key genes (C7, HOOK1, PKP3, AHR, TUFM, GJB1, GSN, MYO6, CLEC7A, and CD74) and 26 miRNA molecules. Specific names of the miRNA molecules are listed in Table S2. TFs combined with NRDEGs were obtained using the ChIPBase and hTFtarget databases. Using Cytoscape software, we structured and visualized a network of mRNA-TF interactions (Fig. 6C). It contains 10 key mRNA genes (C7, HOOK1, PKP3, AHR, TUFM, GJB1, GSN, MYO6, CLEC7A, and CD74) and 100 transcription factors. Specific TF molecule names are listed in Table S3. Finally, CTD database was used to identify potential drugs or molecular compounds of the NRDEGs. The mRNA-drug network was constructed and visualized using Cytoscape software (Fig. 6D), which contained 10 mRNA key genes (C7, HOOK1, PKP3, AHR, TUFM, GJB1, GSN, MYO6, LEC7A, and CD74) and 41 drugs or molecular compounds. The names of specific drugs or molecular compounds are listed in Table S4.

### Expression differences, chromosomal localization, and functional similarity analysis of NRDEGs

**Fig. 7:**
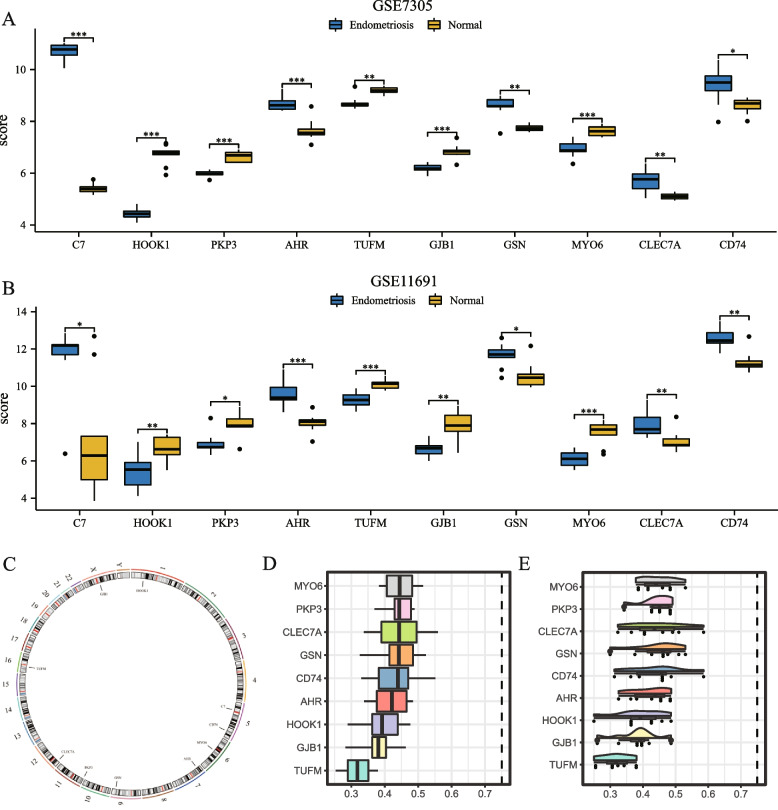
Expression differential analysis, chromosomal localization analysis, and functional similarity analysis of NRDEGs are demonstrated. Group comparison of NRDEGs expression differential analysis results in datasets GSE7305 (**A**) and GSE11691 (**B**). (**C**) Display of chromosome localization results of NRDEGs. Functional similarity analysis results of NRDEGs are shown in the bar chart (**D**) and rain-cloud chart (**E**). * represents *P* value < 0.05, which is statistically significant; ** represents *P* value < 0.01, which is highly statistically significant; *** represents *P* value < 0.001, which is highly statistically significant. NRDEGs, necroptosis-related differentially expressed genes

To further verify the expression difference of NRDEGs in the endometriosis dataset, 10 NRDEGs (C7, HOOK1, PKP3, AHR, TUFM, GJB1, GSN, MYO6, CLEC7A, and CD74) were compared between the groups (Figs. 7A and B). CD74 expression analysis was performed for GSE7305 and GSE11691 in the endometriosis and normal groups, respectively. The difference in the results of dataset GSE7305 (Fig. 7A) demonstrated that all NRDEGs were statistically significant, and the expression levels of C7, HOOK1, PKP3, AHR, GJB1, and MYO6 in different groups of endometriosis dataset GSE7305 were statistically significant (*P* < 0.001). There was a highly statistically significant difference in TUFM, GSN, and CLEC7A expression levels among the groups (*P* < 0.01). The expression of CD74 in the different groups was statistically significant (*P* value < 0.05). The difference results of dataset GSE11691 (Fig. 7B) illustrated that all NRDEGs were statistically significant: AHR, TUFM, and MYO6 expression levels in different groups of dataset GSE76885 were statistically significant (*P* value < 0.001). HOOK1, GJB1, CLEC7A, and CD74 expressions in the different groups were statistically significant (*P* < 0.01). The expression levels of C7, PKP3, and GSN in the different groups were statistically significant (*P* value < 0.05). We then mapped the chromosomal locations of the 10 NRDEGs (C7, HOOK1, PKP3, AHR, TUFM, GJB1, GSN, MYO6, CLEC7A, and CD74) (Fig. 7C). The results depicted that genes C7 and CD74 were placed on chromosome 5, HOOK1 gene was placed on chromosome 1, MYO6 gene was placed on chromosome 6, AHR gene on chromosome 7, GSN gene on chromosome 9, and PKP3 gene on chromosome 11. CLEC7A was placed on chromosome 12, and TUFM was placed on chromosome 12. Based on these scores, scholars analyzed the genes involved in endometriosis lesions and displayed them as bar graphs (Fig. 7D) and rain-cloud graphs (Fig. 7E). The results depict that PKP3, GSN, MYO6, CLEC7A, and CD74 play important roles in this process.

### Immune infiltration analysis

**Fig. 8:**
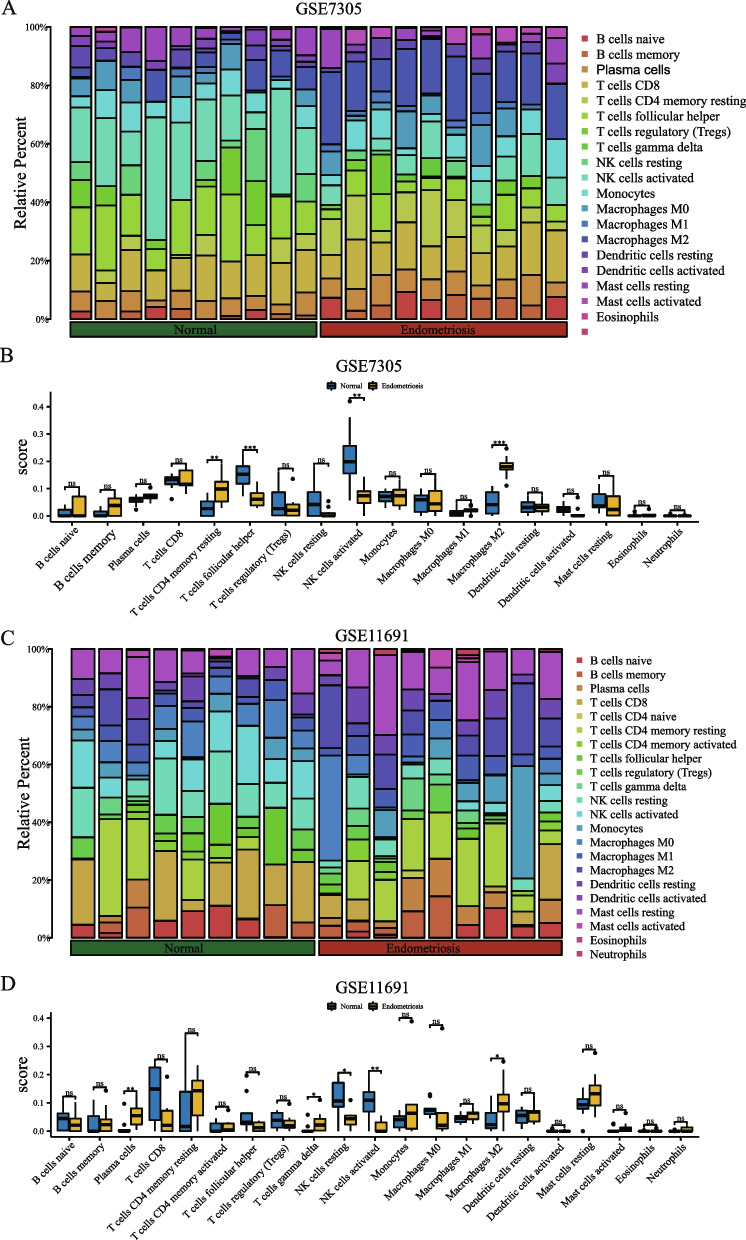
Endometriosis analysis of immune infiltration in GSE7305 and GSE11691 datasets (CIBERSORTx). Results of 22 types of immune cell infiltration in GSE7305 dataset are displayed in the bar chart (**A**) and the group comparison chart (**B**). Results of 22 immune cell infiltration in the GSE11691 dataset are shown in the bar chart (**C**) and the group comparison chart (**D**). The symbol NS means *P* ≥ 0.05, which is not statistically significant. The symbol * is equivalent to *P* < 0.05, which is statistically significant; The symbol ** is equivalent to *P* < 0.01, which is highly statistically significant; The symbol *** is equivalent to *P* < 0.001, which is highly statistically significant

**Fig. 9:**
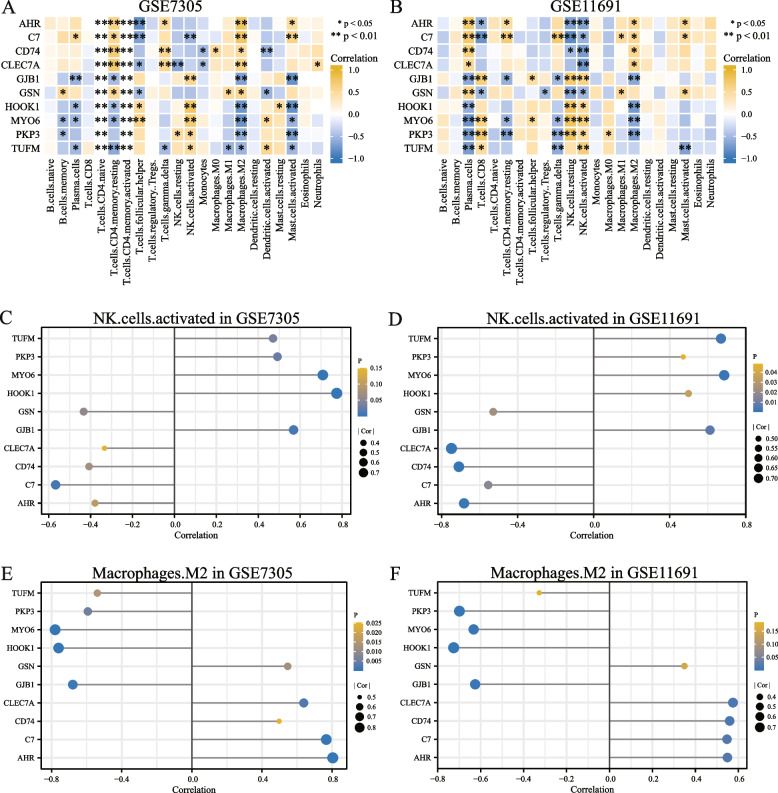
Heatmap of correlation presents immune infiltration results of GSE7305 and GSE11691 datasets and correlation analysis between two kinds of immune cells and NRDEGs. Heatmap showing the correlation of 22 immune cell infiltration results in GSE7305 dataset (**A**) and GSE11691 dataset (**B**). Correlation analysis between immune cells activated Nk. cells and NRDEGs in GSE7305 (**C**) and GSE11691 (**D**) datasets. M2 Macrophages show the correlation between NRDEGs and GSE7305 (**E**) and GSE11691 (**F**). The Y-axis of the lollipop figure represents the specific gene, and the X-axis represents the correlation size. The circle size in the lollipop graph represents the correlation degree; the higher the degree of correlation, the larger the circle, and the different colors of the circle represent the *P* value obtained by the statistical correlation method. The higher the bar (distance from 0), the higher the degree of correlation (positive numbers mean positive correlations, negative numbers mean negative correlations). The symbol * is equivalent to *P* < 0.05, statistically significant; the symbol ** is equivalent to *P* < 0.01, which is highly statistically significant. NRDEGs, necroptosis-related differentially expressed genes

**Fig. 10:**
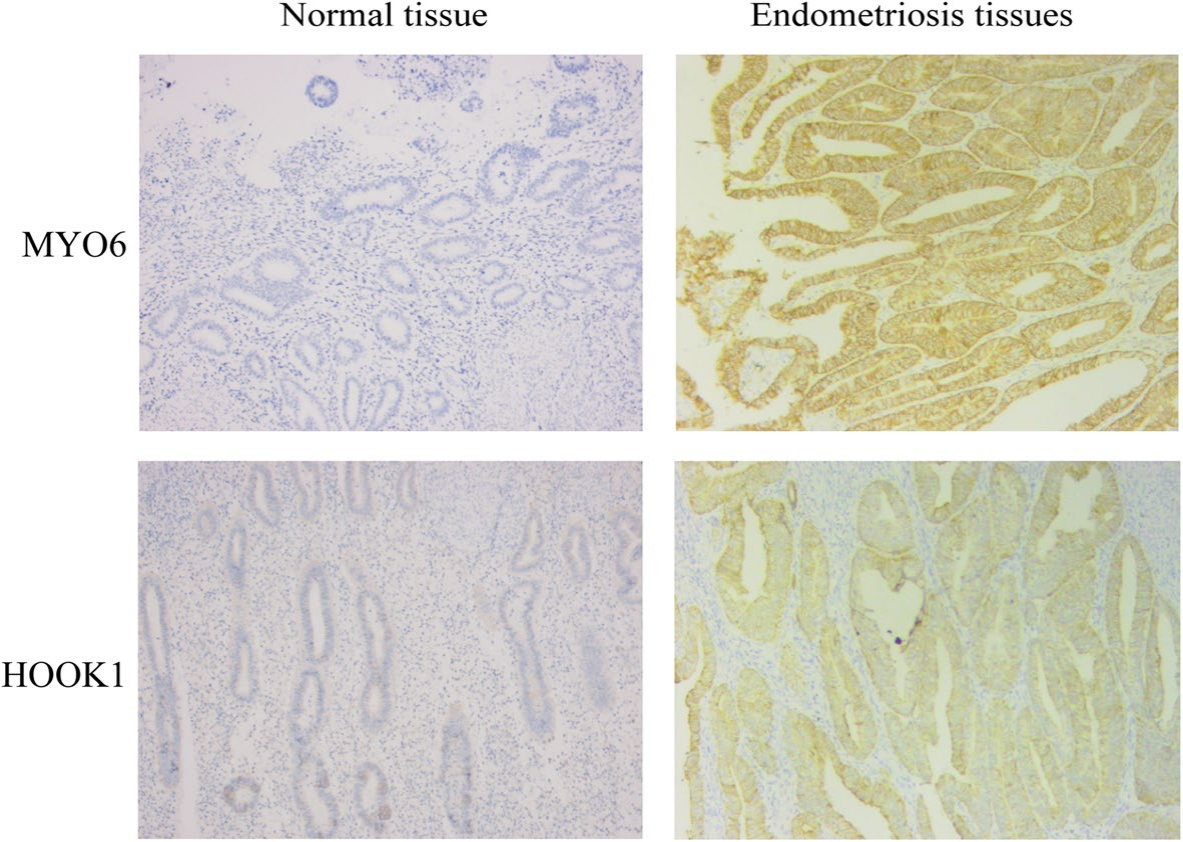
The immunohistochemical result of endometriosis tissue (× 100)

We sorted out the expression profile data of GSE7305 and GSE11691 in the endometriosis dataset and uploaded it to the CIBERSORTx online website. The CIBERSORTx algorithm was used to calculate 22 immune cells and endometriosis samples in the endometriosis dataset (group: endometriosis) and the expression profile data of normal samples (group: normal). Based on the immune infiltration analysis results, we plotted the immune cell infiltration of each sample of the 22 types of immune cells in GSE7305 and GSE11691 datasets in bar graphs (Figs. 8A and C). We also presented group comparison maps to illustrate the correlation between immune cell infiltration abundance and different groups in GSE7305 and GSE11691 datasets (Figs. 8B and D). We demonstrated a correlation between the abundance of six immune cell infiltrates in GSE7305 and GSE11691 datasets using correlation heatmaps (Figs. 9A and B). The results demonstrated that after excluding the immune cells that had insignificant difference after grouping, there were statistically significant differences in the infiltration abundance of four types of immune cells in the GSE7305 dataset (Fig. 8B) and the correlation between samples in different groups (*P* < 0.05). These four immune cells are resting memory CD4 + T cells, follicular helper T-cells, activated NK cells, and M2 Macrophages. In dataset GSE11691 (Fig. 8D), there were five types of immune cells, and the correlation between infiltration abundance and samples in different groups was statistically different (*P* < 0.05). These five immune cells are plasma cells, gamma-delta T cells, resting NK cells, activated NK cells, and M2 Macrophages. The numbers of activated NK cells and M2 Macrophages were statistically significant in both datasets. To analyze the correlation between the expression levels of 10 NRDEGs (C7, HOOK1, PKP3, AHR, TUFM, GJB1, GSN, MYO6, CLEC7A, and CD74) in endometriosis datasets GSE7305, GSE11691 with infiltration abundance of two immune cells (activated NK cells and M2 macrophages). We demonstrated the infiltration abundance of two immune cells (activated NK cells and M2 macrophages) and 10 NRDEGs (C7, HOOK1, PKP3, AHR, TUFM, GJB1, GSN, MYO6, CLEC7A, and CD74) by lollipop figure (Figs. 9C–F). As can be seen from the figure, the corresponding gene MYO6 had high correlation and consistency in the two datasets (*r* = 0.708 in GSE7305, *P* < 0.001; GSE11691 r = 0.686, *P* < 0.001), while M2 Macrophages had higher correlation and consistency in the two datasets for the corresponding genes HOOK1 (*r* = -0.760 in GSE7305, *P* < 0.001; *r* = -0.726 in GSE11691, *P* < 0.001), GJB1 (in GSE7305) *r* = -0.679, *P* < 0.001; *r* = -0.626, *P* < 0.01 in GSE11691), and MYO6 (*r* = -0.780, *P* < 0.001 in GSE7305; *r* = -0.633, *P* < 0.01 in GSE11691). The immunohistochemical result indicated that the protein levels of MYO6 and HOOK1 were increased in patients with endometriosis, further suggesting that MYO6 and HOOK1 can be used as potential biomarkers for endometriosis (Fig. 10).

## Discussion

Most gynecologists detect ovarian endometriosis using laparoscopy, which is the most common form of endometriosis. Recently, microarray and high-throughput sequencing technologies have enabled bioinformatic analysis of endometriosis. However, most studies are based on invasive methods and single arrays, resulting in poor acceptance and a lack of cohorts for multiple combined studies. Its goal is to discover new diagnostic methods and safe treatments for endometriosis by exploring its biological mechanisms and identifying meaningful molecular markers. Therefore, we analyzed patients with and without endometriosis and performed an enrichment analysis of necroptosis-related genes to determine their role in endometriosis.

Disease onset and progression are associated with necroptosis, according to increasing research. Necroptosis, programmed necrotic cell death, is vital for the host's defense against certain pathogen incursions. Inflammatory diseases result from the deregulation of necroptosis [5]. However, its role in the immune response to endometriosis remains unclear. In this study, there were 330 DEGs between 19 endometriosis samples and 19 normal samples in two expression profile datasets (GSE7305 and GSE1169). We intersected the common differentially expressed genes (co-DEGs) and necroptosis-related genes in two endometriosis datasets to obtain 10 NRDEGs from endometriosis datasets. The ten NRDEGs were C7, HOOK1, PKP3, AHR, TUFM, GJB1, GSN, MYO6, CLEC7A, and CD74. Among these, PKP3, which belongs to the plakophilin family, promotes tissue integrity. Plakophilins link desmosomal cadherins to intermediate filaments at desmosome junctions, and in common with other catenins, they perform additional functions, including in the nucleus [16]. Gene GJB1 encodes the transmembrane channel protein connexin 32 (Cx32), a member of the Cxs family [39]. Previous studies have disclosed that GJB1 exerts anti-apoptotic and pro-tumor effects by interacting with it [40, 41]. HOOK1, encodes a member of the hook family of coiled-coil proteins that bind to microtubules and organelles via their N-and C-terminal domains, respectively. In the present study, HOOK1 expression was upregulated. Notably, the N-terminal segment of Hook1 has a cytoskeletal protein-binding site that is involved in cell migration and intracellular vesicle trafficking [42]. The mitochondrial translation elongation factor is encoded by the TUFM. Cho et al. suggested that TUFM might be important for CASP8 inhibition via autophagy activation [43]. Actin-based myosins (MYO6) move cargo towards the minus ends of actin filaments using their actin-based motor proteins. As it is the only myosin with this directionality, it is vital in many biological processes [44]. MYO6 is involved in various physiological processes in vivo, and its expression has been reported to be increased in humans and mice in different diseases, including cancer and hearing loss [45, 46]. The enrichment analysis included GO terms for functional enrichment and GSEA enrichment. In addition to being enriched in immune response and activation, these genes were significantly associated with immune-cell interactions.

In recent decades, accumulating evidence has demonstrated immune imbalance in endometriosis [47]. Compared to normal endometrium, endometriosis patients have more immune cells, particularly NK and M2 cells. However, the immune cell activation pattern in endometriosis remains unclear. The role of immune cell infiltration in endometriosis needs to be further investigated, and we performed a comprehensive evaluation of immune cell infiltration in endometriosis using CIBERSORT. Our study demonstrated a correlation between the infiltration abundance of NK cells and M2 macrophage immune cells. Moreover, genes were statistically different between the GSE7305 and GSE1169 datasets. Previous studies have reported downregulation of NK cells in endometriosis patients; our results are consistent with theirs [48, 49]. Lagana AS et al. [50] found that the number of M1 and M2 macrophages was significantly higher in the endometriosis group than in controls, regardless of stage. Moreover, M2 macrophages may inhibit the immunological response of NK cells. Reduced NK cell number and function result in reduced cytotoxicity and elimination of ectopic endometrial cells [51, 52]. In our study, the relative genes of NK cell activation were MYO6 and macrophages. M2 represents HOOK1 expression in the two datasets. MYO6 and HOOK1 have a limited impact on the immune system in endometriosis. A macromolecular antigen, ovalbumin, showed high permeability to Rmc monolayers lacking myo6. It still induced strong T-cell activation because it retained antigenicity. In a study by Yu-wei Liao et al. [53], MyO6-deficient RMC monolayers demonstrated high permeability, retaining ovalbumin antigenicity, thereby activating T cells. Our findings suggest that MYO6 and HOOK1 are associated with immune infiltration in endometriosis and can be used as novel potential biomarkers and predictors of immune cell infiltration in endometriosis.

Necroptosis represents the newly discovered immunogenic cell death (ICD) forms. Evidence shows that necroptosis modulates the immune system, primarily composed of natural killer (NK) cells, macrophages, dendritic cells (DC), and T and B lymphocytes [54]. To investigate whether MYO6 and HOOK1 contribute to immune cell infiltration, we explored the correlation between these two factors. Necroptosis and the immune response appear to be interconnected in endometriosis, as indicated by their significant association with immune cells. The molecular mechanisms underlying the complex interactions between these genes and immune cells should be elucidated in future studies. Molecular classification can improve risk stratification and management of EC [55, 56]. Hence, the identification and verification of molecular classification in endometriosis, as well as the examination of diverse molecular markers, have the potential to significantly impact the treatment approach, particularly for patients with fertility requirements, by modifying the surgical methodology and altering the follow-up strategy. It has been depicted to correlate radiological features with molecular/genomic profiles to classify endometrial cancer prognosis [57]. Ultrasound serves as a prevalent diagnostic tool for endometriosis, followed by the integration of radiomics with molecular/genomic profiling, enabling the tailoring of surgical and post-surgical treatment approaches for patients with endometriosis. In the era of personalized medicine, it is necessary to determine the best treatment for each patient with endometrial cancer based on the patient's molecules/genes [58]. In the foreseeable future, the implementation of molecular/genomic analysis will facilitate the tailoring of the optimal therapeutic approach for endometriosis. With the current understanding of the molecular mechanisms of endometrial disease, discussing the results of this study may contribute to future directions in endometriosis treatment and management.

This study identified 26 miRNAs associated with NRDEGs using the StarBase and miRDB databases. In endometriosis, miRNAs are associated with genetic, epigenetic, and angiogenic factors, hormones, cytokines, chemokines, oxidative stress (OS) markers, inflammation mediators, hypoxia, angiogenesis, and an altered immune system, which contribute to its pathogenesis [59]. A total of 100 transcription factors (TFS) combined with NRDEGs were obtained from the ChIPBase and hTFTarget databases. Previous studies have demonstrated that miR-106a-5p inhibits the proliferation, migration, and invasion of ectopic endometrial stromal cells by targeting the forkhead box transcription factor FOXC1 via the PI3K/Akt/mTOR signaling pathway [60]. Due to the lack of effective therapeutic drugs for EMS [61] based on the CTD database, 41 molecular compounds and drugs that are potentially effective against EM were identified by NRDEGs.

Our study has some limitations. First, we conducted a comprehensive bioinformatic analysis to identify the association between NRDEGs and endometriosis. Further in vitro and in vivo studies are needed to validate the role of necroptotic-related genes in endometriosis and gain a deeper understanding of its pathogenesis. Second, this was a retrospective study; therefore, important clinical information could not be obtained. Third, the specimens in this study were from endometriotic tissue; therefore, the biomarkers could not be used to diagnose the early stages of the disease, and more research on blood biomarkers is required.

## Conclusions

Ten NRDEGs (C7, HOOK1, PKP3, AHR, TUFM, GJB1, GSN, MYO6, CLEC7A, and CD74) may serve as diagnostic biomarkers for endometriosis have been found for the first time. MYO6 and HOOK1 can be used as potential biomarkers for endometriosis. A strong association was also found between the two selected genes and immune cell infiltration to explore the pathogenesis of endometriosis, which could provide a rationale for future treatments. These findings increase our knowledge of necroptosis genes in EMS patients. However, the role of these necroptosis genes in EMS requires validation in the future.

### Supplementary Information

**Additional file 1: Table S1. **List of necroptosis-related genes.

**Additional file 2: Table S2. **List of miRNA of mRNA–miRNA network.

**Additional file 3: Table S3. **List of TF of mRNA–TF network.

**Additional file 4: Table S4. **List of drugs of mRNA–drug network.

## Data Availability

The datasets generated and/or analysed during the current study are available in the OSF repository, https://osf.io/skgpe/. Further inquiries can be directed to the corresponding authors.
