# Supplementary material for: Signatures of necroptosis-related genes as diagnostic markers of endometriosis and their correlation with immune infiltration
Source: BMC Womens Health. 2023 Oct 11;23:535. doi: 10.1186/s12905-023-02668-7 (PMC10566087; doi:10.1186/s12905-023-02668-7)
Supplement: Supplementary file 2 — Additional file 2: Table S2. List of miRNA of mRNA–miRNA network. [file 12905_2023_2668_MOESM2_ESM.docx]

Table S2. List of miRNA of mRNA–miRNA network.

| mRNA | miRNA |
| --- | --- |
| AHR | hsa-miR-380-3p |
| AHR | hsa-miR-374a-5p |
| AHR | hsa-miR-379-3p |
| AHR | hsa-miR-411-3p |
| AHR | hsa-miR-374b-5p |
| AHR | hsa-miR-186-5p |
| AHR | hsa-miR-3163 |
| AHR | hsa-miR-124-3p |
| AHR | hsa-miR-142-5p |
| AHR | hsa-miR-506-3p |
| AHR | hsa-miR-381-3p |
| AHR | hsa-miR-300 |
| AHR | hsa-miR-5590-3p |
| AHR | hsa-miR-126-5p |
| AHR | hsa-miR-433-3p |
| AHR | hsa-miR-576-5p |
| AHR | hsa-miR-577 |
| AHR | hsa-miR-340-5p |
| AHR | hsa-miR-3679-5p |
| AHR | hsa-miR-1185-5p |
| AHR | hsa-miR-302e |
| AHR | hsa-miR-96-5p |
| AHR | hsa-miR-1271-5p |
| AHR | hsa-miR-522-3p |
| AHR | hsa-miR-542-3p |
| C7 | hsa-miR-21-5p |
| C7 | hsa-miR-510-5p |
| C7 | hsa-miR-590-5p |
| CD74 | hsa-miR-4731-5p |
| CD74 | hsa-miR-320b |
| CD74 | hsa-miR-320c |
| CD74 | hsa-miR-320d |
| CD74 | hsa-miR-4429 |
| GSN | hsa-miR-124-3p |
| GSN | hsa-miR-450b-5p |
| GSN | hsa-miR-141-3p |
| GSN | hsa-miR-200a-3p |
| GSN | hsa-miR-506-3p |
| HOOK1 | hsa-miR-216a-5p |
| HOOK1 | hsa-miR-200b-3p |
| HOOK1 | hsa-miR-200c-3p |
| HOOK1 | hsa-miR-429 |
| HOOK1 | hsa-miR-576-5p |
| HOOK1 | hsa-miR-218-5p |
| HOOK1 | hsa-miR-2115-3p |
| HOOK1 | hsa-miR-223-3p |
| HOOK1 | hsa-miR-361-5p |
| HOOK1 | hsa-miR-543 |
| HOOK1 | hsa-let-7a-5p |
| HOOK1 | hsa-let-7b-5p |
| HOOK1 | hsa-let-7c-5p |
| HOOK1 | hsa-let-7d-5p |
| HOOK1 | hsa-let-7e-5p |
| HOOK1 | hsa-let-7f-5p |
| HOOK1 | hsa-miR-98-5p |
| HOOK1 | hsa-let-7g-5p |
| HOOK1 | hsa-let-7i-5p |
| HOOK1 | hsa-miR-4458 |
| HOOK1 | hsa-miR-4500 |
| MYO6 | hsa-miR-145-5p |
| MYO6 | hsa-miR-5195-3p |
| MYO6 | hsa-miR-143-3p |
| MYO6 | hsa-miR-1323 |
| MYO6 | hsa-miR-4770 |
| MYO6 | hsa-miR-548o-3p |
| MYO6 | hsa-miR-6088 |
| MYO6 | hsa-miR-770-5p |
| MYO6 | hsa-miR-4712-5p |
| MYO6 | hsa-miR-579-3p |
| MYO6 | hsa-miR-664b-3p |
| MYO6 | hsa-miR-589-5p |
| MYO6 | hsa-miR-494-3p |
| MYO6 | hsa-miR-766-5p |
