# Supplementary material for: Signatures of necroptosis-related genes as diagnostic markers of endometriosis and their correlation with immune infiltration
Source: BMC Womens Health. 2023 Oct 11;23:535. doi: 10.1186/s12905-023-02668-7 (PMC10566087; doi:10.1186/s12905-023-02668-7)
Supplement: Supplementary file 4 — Additional file 4: Table S4. List of drugs of mRNA–drug network. [file 12905_2023_2668_MOESM4_ESM.docx]

Table S4. List of drugs of mRNA–drug network.

| mRNA | drug |
| --- | --- |
| C7 | Copper Sulfate |
| C7 | Diuron |
| HOOK1 | Valproic Acid |
| HOOK1 | Tetrachlorodibenzodioxin |
| HOOK1 | Acetaminophen |
| HOOK1 | methylmercuric chloride |
| HOOK1 | 1,2-Dimethylhydrazine |
| PKP3 | Tetrachlorodibenzodioxin |
| PKP3 | Benzo(a)pyrene |
| PKP3 | sodium arsenite |
| PKP3 | Thioacetamide |
| PKP3 | Valproic Acid |
| PKP3 | 2,4-dinitrotoluene |
| AHR | Tetrachlorodibenzodioxin |
| AHR | alpha-naphthoflavone |
| AHR | Benzo(a)pyrene |
| AHR | beta-Naphthoflavone |
| AHR | Methylcholanthrene |
| AHR | Resveratrol |
| AHR | 3,4,5,3',4'-pentachlorobiphenyl |
| AHR | 2-methyl-2H-pyrazole-3-carboxylic acid (2-methyl-4-o-tolylazophenyl)amide |
| AHR | Phenobarbital |
| TUFM | Cyclosporine |
| TUFM | pirinixic acid |
| TUFM | Valproic Acid |
| GJB1 | Acetaminophen |
| GJB1 | Diethylnitrosamine |
| GJB1 | Benzo(a)pyrene |
| GJB1 | Carbon Tetrachloride |
| GJB1 | Aflatoxin B1 |
| GJB1 | Cyclosporine |
| GJB1 | Atrazine |
| GJB1 | Paraquat |
| GJB1 | Urethane |
| GJB1 | Hexachlorobenzene |
| GJB1 | ochratoxin A |
| GJB1 | Propylthiouracil |
| GJB1 | Tetrachlorodibenzodioxin |
| GSN | Valproic Acid |
| GSN | Tetrachlorodibenzodioxin |
| GSN | Aflatoxin B1 |
| GSN | Tretinoin |
| GSN | Cyclosporine |
| GSN | bisphenol A |
| GSN | Troglitazone |
| MYO6 | Valproic Acid |
| MYO6 | Tetrachlorodibenzodioxin |
| MYO6 | Acetaminophen |
| MYO6 | Benzo(a)pyrene |
| MYO6 | bicalutamide |
| MYO6 | bisphenol A |
| CLEC7A | Lipopolysaccharides |
| CLEC7A | Tetrachlorodibenzodioxin |
| CLEC7A | bisphenol A |
| CLEC7A | Carbon Tetrachloride |
| CLEC7A | titanium dioxide |
| CLEC7A | Formaldehyde |
| CD74 | Tetrachlorodibenzodioxin |
| CD74 | pirinixic acid |
| CD74 | Tobacco Smoke Pollution |
| CD74 | Doxorubicin |
| CD74 | Ethinyl Estradiol |
| CD74 | trichostatin A |
| CD74 | Vehicle Emissions |
| CD74 | Benzo(a)pyrene |
